# Probing Asymmetric Interactions with Time-Separated Mutual Information: A Case Study Using Golden Shiners

**DOI:** 10.3390/e26090775

**Published:** 2024-09-10

**Authors:** Katherine Daftari, Michael L. Mayo, Bertrand H. Lemasson, James M. Biedenbach, Kevin R. Pilkiewicz

**Affiliations:** 1 Department of Mathematics, University of North Carolina at Chapel Hill, Chapel Hill, NC 27599, USA; 2 U.S. Army Engineer Research and Development Center, 3909 Halls Ferry Road, Vicksburg, MS 39180, USA; michael.l.mayo@erdc.dren.mil (M.L.M.); bertrand.h.lemasson@usace.army.mil (B.H.L.); james.m.biedenbach@usace.army.mil (J.M.B.)

**Keywords:** information theory, collective motion, animal behavior

## Abstract

Leader–follower modalities and other asymmetric interactions that drive the collective motion of organisms are often quantified using information theory metrics like transfer or causation entropy. These metrics are difficult to accurately evaluate without a much larger number of data than is typically available from a time series of animal trajectories collected in the field or from experiments. In this paper, we use a generalized leader–follower model to argue that the time-separated mutual information between two organism positions can serve as an alternative metric for capturing asymmetric correlations that is much less data intensive and more accurately estimated by popular *k*-nearest neighbor algorithms than transfer entropy. Our model predicts a local maximum of this mutual information at a time separation value corresponding to the fundamental reaction timescale of the follower organism. We confirm this prediction by analyzing time series trajectories recorded for a pair of golden shiner fish circling an annular tank.

## 1. Introduction

For many groups of social organisms, collective motion tends to be precipitated by one or more “leaders’’ who act first in response to a stimulus (sensing a food source, detecting an encroaching predator, etc.) and seemingly drive the rest of the group to follow their example. Seemingly is the proper qualifier, because, although numerous studies have attempted to quantify the collective benefits of leader–follower dynamics [1,2,3,4] and identify behavioral features that make certain organisms more likely to take the lead in different scenarios [5,6,7,8], it is rather difficult to prove that the organisms who act later are actually being influenced by the leader and are not merely making independent, albeit delayed, decisions [9,10]. Indeed, one can think of leader–follower behavior as a continuum, with organisms that blindly follow the leader at one extreme, and those that make independent decisions simply coinciding with the leader’s at the other.

To illustrate this latter extreme, consider a group of runners competing in a race. At any moment after the starting gun, one individual will be in the lead, and the other competitors will apparently be following this leader; but the reality, of course, is that each runner moves independently toward the finish line while jockeying for lead position. In this example, we understand that a true leader–follower modality is not playing out based on our contextual knowledge of the situation, but how is one to make a similar determination from observations of a flock of ducks flying in their characteristic v-formation or a mass of ants marching in single file?

At minimum, a leader–follower interaction must be asymmetric and retarded in time: the leader must influence the motion of the follower more strongly than vice versa, and the follower cannot react instantaneously to the motion of the leader. These requirements suggest that a necessary (but not sufficient) condition for the existence of a leader–follower relationship is a disparity in the size of the correlations between the present motion of one individual with the past motion of the other. The correlations should be large when comparing the present motion of the putative follower with the past motion of the presumed leader and should be small (ideally near zero) in the opposite case.

Currently, one of the most prominent metrics used to quantify these types of correlations is transfer entropy [11,12,13,14]. For a pair of discrete-time Markov chains, *X* and *Y*, the transfer entropy from process *Y* to process *X* is defined as [15]:

(1) $$\begin{array}{cc} TE_{Y\to X} & =\sum_{x_{t}}\sum_{x_{t-1}}\sum_{y_{t-1}}p\left(x_{t},x_{t-1},y_{t-1}\right)\\ \; & \times \log\left[\frac{p(x_{t},y_{t-1}|x_{t-1})}{p\left(x_{t}|x_{t-1}\right)p\left(y_{t-1}|x_{t-1}\right)}\right], \end{array}$$

where $x_{t}$ is the value of the Markov process, *X*, at time *t*, denoted $X_{t}$, and $p(x_{t})$ is its probability distribution function. This transfer entropy measures the amount of information shared between $X_{t}$ and $Y_{t-1}$, given that $X_{t-1}$ is known. If we understand the conditioned random variable $X_{t}|X_{t-1}$ as encoding the stochastic dynamics of process *X*, i.e., how the process chooses its value at time *t*, given its previous value, then $TE_{Y\to X}$ quantifies how much the dynamics of *X* are informed by the past state of *Y*.

Though popular, transfer entropy has a number of shortcomings that make it difficult for it to accurately compute from experimental datasets. Most obviously is the fact that the trajectories of living organisms are not discrete-time Markov chains. For discrete stochastic processes X(t) and Y(t) that have finite memories of *ℓ* and *m* time steps, respectively, Equation (1) can be generalized by replacing $X_{t-1}$ and $Y_{t-1}$ with the sets $\left\{X_{t-1},\ldots ,X_{t-l}\right\}$ and $\left\{Y_{t-1},\ldots ,Y_{t-m}\right\}$ [16]. There are even ways of generalizing the notion of transfer entropy to continuous time systems with a finite memory [17,18], but any sort of conditioning over a larger number of past states necessitates numerically estimating a higher-dimensional joint distribution. This is less of an issue for an in silico model that can be simulated for arbitrarily long periods of time to produce datasets that representatively sample this high-dimensional multivariate distribution; but for experimental measurements of animal motion that often consist of a limited number of short time series, it is a serious problem [19]. One common solution is to determine a mesoscopic timescale, Δt, over which the dynamics of the experimental system are approximately Markovian, and then compute the transfer entropy by replacing t−1 in Equation (1) with t−Δt. Again, however, data scarcity tends to preclude a rigorous estimation of this time interval, so often one settles for selecting a timescale that is known to be fundamental to the motion of the organisms under consideration [11]. The hope is that this physically motivated timescale will be sufficient to capture any asymmetry in the time-delayed influence between two organisms to determine whether a leader–follower type interaction between them is likely to exist.

Because of the higher-dimensional joint probabilities involved, accurately estimating the transfer entropy is a nontrivial task. The *k*-nearest neighbors estimator for mutual information first proposed by Kraskov, Stögbauer, and Grassberger [20] (henceforth abbreviated as the KSG estimator) has become the standard in many information theory studies [21,22,23]; but, although the algorithm can be straightforwardly generalized to estimate transfer entropy, it has been shown that this generalization reduces the accuracy of the method [24]. Alternative estimation schemes do exist, such as the use of kernel density estimators, but comparative studies have found them to perform, at best, only marginally better than the KSG method [25,26].

There are, of course, other issues with using transfer entropy to infer causality. For example, if two agents described by the stochastic processes X(t) and Y(t) are both following the same leader agent, described by the process Z(t), the transfer entropy $TE_{Y\to X}$ will typically be nonzero, even though follower *Y* is not directly influencing follower *X*. The transfer entropy $TE_{Z\to X}$ will usually exceed $TE_{Y\to X}$, but this quantitative difference can sometimes be hard to characterize with statistical significance from short and noisy time series. Metrics such as causation entropy [27,28] attempt to correct for this deficiency in distinguishing direct from indirect influence, but these metrics require the estimation of even higher dimensional multivariate distributions, compounding the computational shortcomings of transfer entropy.

In this paper, we argue for the use of time-separated mutual information as a viable alternative to transfer entropy for the purposes of identifying leader–follower interactions between pairs of organisms. For the Markov processes *X* and *Y* defined previously, this mutual information is defined as follows:

(2) $$\begin{array}{cc} MI(X_{t};Y_{t-\Delta t}) & =\sum_{x_{t}}\sum_{y_{t-\Delta t}}p\left(x_{t},y_{t-\Delta t}\right)\\ \; & \times \log\left[\frac{p(x_{t},y_{t-\Delta t})}{p\left(x_{t}\right)p\left(y_{t-\Delta t}\right)}\right] \end{array}$$

The principal argument against using this metric to characterize directed interactions is that it merely tells us whether the present state of process *X* is correlated with the past state of process *Y*, *not* whether the dynamics of *X* are causally influenced by *Y*. Numerous illustrative examples have been worked out in the literature to support this assertion [15,17,29]; indeed, our earlier footrace example is one where the mutual information of Equation (2), for a fixed Δt, would likely be substantial in value regardless of whether the second-place runner is actively pursuing the lead runner or merely coincidentally moving towards the same destination. We will argue, however, that, *as a function of Δt*, the time-separated mutual information will exhibit a markedly asymmetric profile in the former case but not the latter. This assertion is consistent with findings in the field of neuroscience, wherein time-separated mutual information has been used to quantify both the direction and timescale of interactions between signaling neurons [30,31]. Studies from fields like genetics [32,33] and control theory [34] have likewise demonstrated the utility of this metric in shedding light on the causal structure of correlated time series data, even in cases where the time points are irregularly and sparsely sampled [35].

We derive this result by considering a highly general model that typifies what a leader–follower type interaction must, at minimum, involve. Regardless of the influence of any competing interactions that enhance the stochastic character of the dynamics, we prove that the time-separated mutual information should exhibit a peak at some characteristic response timescale Δt=T and decay monotonically as |Δt−T|→∞ towards some asymptotic value. For a typical experimental dataset, consisting of the time series trajectories for a pair of organisms, the predicted peak in the time-separated mutual information may not exceed its asymptotic value by a statistically significant amount, which could indicate either the complete lack of a leader–follower mechanism or the presence of other, dominant interactions that wash out the leader–follower contribution to the dynamics. If a statistically significant peak is observed, on the other hand, then we can at least assert that the behavior of the organisms can be consistently explained by a simple leader–follower-type mechanism.

After analyzing our generalized leader–follower model, we proceed to a discussion of the formal requirements for correctly computing statistical metrics from a single time series. Carefully designed experiments are the key to producing data fulfilling these criteria, and we detail how we implement these desirable features in designing a set of experiments in which we record the motion of pairs of golden shiner fish (*Notemigonus crysoleucas*) swimming around an annular tank. For a well-behaved trajectory in which the two fish swim smooth, staggered laps around their tank, we use the time-separated mutual information between the angular positions of the two fish to demonstrate that their fluctuating angular separation is consistent with a simplified version of our general leader–follower model, and we relate the emergent response timescale to the known visual reaction timescale of these fish.

## 2. Materials and Methods

### 2.1. Generalized Leader–Follower Model

In this section, we wish to encode our understanding of what a leader–follower interaction should minimally entail into a simple yet generalizable modeling framework. To that end, we consider two agents—a leader and a follower—whose configurations are, respectively, described by the vectors of random variables ${\vec{X}}_{L}\left(t\right)$ and ${\vec{X}}_{F}\left(t\right)$. For point particle agents, their configuration is simply their vector position; for more realistic agents, the configuration may also include elements such as orientational coordinates, deformation coordinates, etc. The key point is that these two vectors evolve in discrete time according to the following pair of iterative equations:

(3) $$\begin{array}{cc} {\vec{X}}_{L}\left(t\right) & ={\vec{X}}_{L}\left(t-1\right)+\Delta {\vec{X}}_{L}\left(t\right)\\ {\vec{X}}_{F}\left(t\right) & ={\vec{X}}_{L}\left(t-T\right)+\vec{\xi }\left(t\right). \end{array}$$

In the above, all times are given in units of an arbitrary (presumably small) time step and $\Delta {\vec{X}}_{L}\left(t\right)$ is a random variable representing the change in the configurational variables ${\vec{X}}_{L}$ between consecutive time steps *t* and t−1. All of the dynamics driving the motion decisions of the leader organism are modeled through $\Delta {\vec{X}}_{L}\left(t\right)$, and we assume nothing about the distribution of this random variable other than that it does not depend explicitly upon the configuration of the follower.

The follower, on the other hand, attempts to pursue the same course as the leader, but its finite reaction time results in it lagging behind by *T* time steps. The follower also cannot tail the leader perfectly, and its tendency to err is quantified by the random vector $\vec{\xi }\left(t\right)$. Again, we assume very little about the distribution of this noise term; it may even include additional deterministic interactions that compete with or distract from its following tendency. For the leader–follower interaction to dominate the dynamics, we only require that both the mean and variance of $\vec{\xi }\left(t\right)$ must be small on the scale of the domain through which the agents are moving.

At first blush, this model seems to exclude many of the complexities found in other self-propelled particle models [36]. For example, other models evolve in continuous time and incorporate inertial effects as well as external forces like hydrodynamics [37,38]; but these effects can in principle be included into the definition of $\Delta {\vec{X}}_{L}\left(t\right)$ by defining it as the temporally discretized solution of a more sophisticated continuous-time particle model, such as that found in reference [39]. Similarly, other deterministic forces between the agents (such as a close-ranged agent–agent repulsive force) can likewise be included as deterministic components of the stochastic variables $\Delta {\vec{X}}_{L}\left(t\right)$ and $\vec{\xi }\left(t\right)$.

We now consider the time-separated mutual information $MI({\vec{X}}_{F}\left(t\right);{\vec{X}}_{L}\left(t-\tau \right))$ as a function of the time separation, τ. We can relate ${\vec{X}}_{L}\left(t-\tau \right)$ to ${\vec{X}}_{L}\left(t-T\right)$ by iterating the first line of Equation (3) either forwards in time or backwards, depending on whether τ is later or earlier than time *T*. The second line of Equation (3) can then be used to relate ${\vec{X}}_{L}\left(t-T\right)$ to ${\vec{X}}_{F}\left(t\right)$ as follows:

(4) $${\vec{X}}_{L}\left(t-\tau \right)=\left\{\begin{array}{cc} {\vec{X}}_{F}\left(t\right)-\vec{\xi }\left(t\right) & \\ \;-\sum_{n=1}^{\tau -T}\Delta {\vec{X}}_{L}\left(t-\tau +n\right), & \tau \geq T\\ {\vec{X}}_{F}\left(t\right)-\vec{\xi }\left(t\right) & \\ \;+\sum_{n=0}^{T-\tau -1}\Delta {\vec{X}}_{L}\left(t-\tau -n\right), & \tau <T \end{array}\right.$$

The above equation enables us to express ${\vec{X}}_{L}\left(t-\tau \right)$ as ${\vec{X}}_{F}\left(t\right)$ plus or minus a sum of terms that, by construction, do not depend upon the configurational state of the follower. This implies that, as |T−τ| increases, the time-separated mutual information must decrease, since each additional term of the form $\Delta {\vec{X}}_{L}\left(t\right)$ simply contributes additional information not shared with ${\vec{X}}_{F}\left(t\right)$. (This monotonic decrease can also be understood as a direct consequence of the data-processing inequality.) This means that, as a function of τ, the time-separated mutual information for models of this class should have a peak value at τ=T and then decay towards some asymptotic value as τ either increases or decreases away from *T*. This asymptotic value will typically be zero for an open system, wherein the agents can drift arbitrarily far from one another; but, for agents limited to moving within a confined geometry, this long-time value will often be nonzero (as we shall demonstrate explicitly in Section 3).

The parameter *T* physically represents an effective signaling timescale quantifying how long it takes information about the motion of the leader to travel to the follower, be processed cognitively, and then produce an observable response [30]. For situations where the transmission of information and cognitive processing can be assumed to be of comparatively negligible duration (such as in the case of visual communication, wherein the information propagates at the speed of light), the quantity *T* can be understood simply as the reaction time of the follower.

If the model is at steady state, then we can exploit time translational invariance to show that the index-swapped mutual information $MI({\vec{X}}_{L}\left(t\right);{\vec{X}}_{F}\left(t-\tau \right))$ can be obtained by reflecting $MI({\vec{X}}_{F}\left(t\right);{\vec{X}}_{L}\left(t-\tau \right))$ across the ordinate axis, i.e., replacing τ with −τ, and then translating both time arguments by τ. In cases where the leadership is uncertain, this formalism allows us to plot the time-separated mutual information for one assumed leader/follower pairing on the τ>0 axis and that of the flipped leader/follower pairing on the τ<0 axis of the same graph. Plotted in this manner, the time-separated mutual information will not be (except in very special cases) a symmetric function about τ=0, making it evident that $MI\left({\vec{X}}_{F}\left(t\right);{\vec{X}}_{L}\left(t-\tau \right)\right)\neq MI\left({\vec{X}}_{L}\left(t\right);{\vec{X}}_{F}\left(t-\tau \right)\right)$, and the inherent asymmetry of the leader–follower dynamics can be adequately captured by this two-point correlative metric. Our choice to refer to this metric as “time-separated” as opposed to “time-delayed”, which dominates in the literature [40,41,42], is precisely because we formally allow τ to be negative.

### 2.2. Generating Statistics from a Single Time Series

Before we discuss the experimental system used to validate our generalized leader–follower model, it is worth discussing some of the difficulties inherent in computing information theory metrics from experimentally generated trajectory data and how designing experiments with these concerns in mind can help mitigate their impact. For a model system that is simulated in silico, it is generally not an issue to produce a large volume of replicate trajectories, each initiated from the same starting distribution of system configurations. For any time *t* after the start of the simulations, one can treat the corresponding time points of each replicate trajectory as statistically independent samples of the underlying configurational distribution at that time point. As long as the number of replicates is large enough to sample this distribution representatively, any statistic of the distribution can be computed accurately.

For trajectory data generated from the recorded motion of real organisms, producing such a high volume of replicates is infeasible, and so one often has no alternative but to treat points taken from the same time series as independent samples. This is only a reasonable assumption under two conditions. First, the system must be at steady state, so that the underlying distribution of configurations is stationary in time. Second, the time points selected as samples must be chosen far enough apart in time that any correlations between them have decayed to zero.

**Figure 1:**
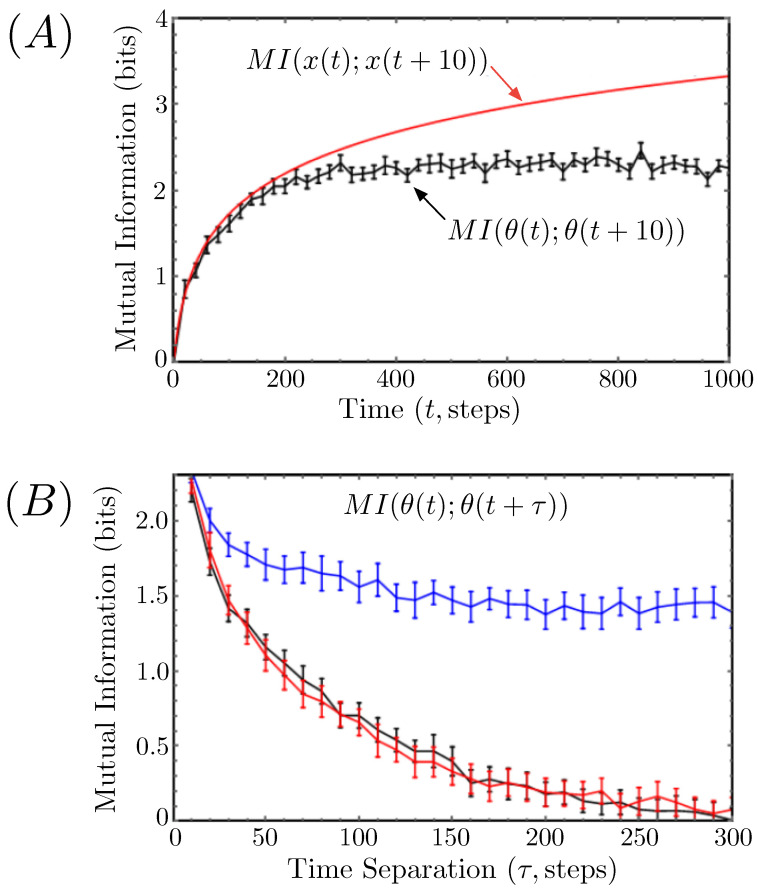
(**A**) The time-separated self mutual information (for fixed time separation τ=10 time steps) of the position of a standard, one-dimensional Gaussian random walker is plotted versus the absolute time *t* (in red). This curve is compared with the same mutual information for a Gaussian random walker confined to the perimeter of a ring of unit radius (in black). The latter is computed numerically from an ensemble of replicate simulations using the KSG algorithm. A jackknifing procedure is used to generate multiple datasets, and the mean information is plotted with standard error bars. (Section 3.2 describes this procedure in more detail.) (**B**) The mutual information for the Gaussian random walker on a ring is plotted at steady state as a function of time separation, τ. The exact information—computed from an ensemble of replicates—is plotted in black. The blue curve is computed from a single trajectory without accounting for the correlations between individually sampled pairs of time points. The red curve is computed from a single trajectory as well, but now each sampled pair of time points is at least 300 time steps apart from every other sample.

Many stochastic processes are inherently nonstationary, even at long times, but one can often force these systems to settle into a long-time steady state by constraining their dynamics to evolve within a finite domain. As a simple example of this, the top (red) curve in Figure 1A plots the mutual information MI(x(t);x(t+τ)) for a one-dimensional Gaussian random walker as a function of time, for fixed time separation τ=10 time steps. This information can be computed analytically and shown to be:

(5) $$MI\left(x(t);x(t+\tau )\right)=\frac{1}{2}\log\left(1+\frac{t}{\tau }\right),$$

where the base of the logarithm, as usual, determines the “units” of the information (base-2 for bits, base-*e* for nats). For any fixed time separation, τ, this information grows logarithmically with time *t*, because the underlying distribution of x(t) has a variance that grows linearly with time as the walker tends to drift further and further from its initial position.

The bottom (black) curve in Figure 1A plots the self mutual information MI(θ(t);θ(t+τ)) for the same Gaussian random walker when confined to the perimeter of a disk of unit radius. Now for fixed time separation, the information plateaus towards a limiting stationary value as t→∞. This is because the finite circumference of the ring limits how far the walker can drift from its starting position. If the walker step size distribution has variance $\sigma^{2}$, then the information should approach a steady state for times much longer than 2π/σ (in the figure, $\sigma^{2}=0.1$). In other words, once the walker has had time to circle its domain many times over, the distribution of its angular position will approach stationarity.

Even if the distribution of configurations is stationary, the underlying stochastic process responsible for evolving one configuration into another will necessarily cause time-separated trajectory points to be nontrivially correlated. Fortunately, these correlations typically decay over some characteristic timescale, so, if one chooses configurations separated by times much longer than that scale, they can be treated, to good approximation, as statistically independent samples of the underlying, stationary distribution.

Once again, using the example of the Gaussian random walk on a ring, Figure 1B plots the steady-state value of the mutual information MI(θ(t);θ(t+τ)) as a function of the time separation, τ, in black. This information was computed numerically using an ensemble of replicate simulations, each of which was allowed to reach steady state before any data were collected. We compare this to the steady-state mutual information obtained from a single trajectory by naively assuming each point in the time series is a statistically independent sample (top curve in the panel, in blue). The much larger information values that result from this calculation are a result of compounding the correlations between the two time points separated by τ in each sample with those that arise between different sampled pairings of points. The former correlations are what we want to measure, and the latter are what we want to exclude.

Based on an estimate of the decay timescale of the ground truth mutual information in Figure 1B, we also compute the mutual information using a subset of a single time series whose pairs of time-separated points are themselves separated from each other by a window of at least 300 time points (for example, if one pair of points is $t_{1}$ and $t_{1}+\tau$, then the next pair, $t_{2}$ and $t_{2}+\tau$, is chosen such that $t_{2}-t_{1}\geq 300$). The resulting curve, plotted in red, is statistically indistinguishable from the ground truth, as desired.

In the following sections we will continue to compare the mutual information estimated from a single time series to that estimated from an ensemble of replicate time series, so it will be useful to adopt a notation to distinguish the two. When we compute the mutual information between two random variables, *X* and *Y*, from a single time series, we will denote this information using the standard notation MI(X;Y). When we compute this same information using an ensemble of replicate time series, we will denote it MI({X};{Y}); we have used brackets in the traditions of set theory to denote that each random variable is sampled from a set of replicate trajectories.

### 2.3. Golden Shiner Experiments

To assess whether the salient features of the time-separated mutual information we derived for our generalized leader–follower model could emerge from an analysis of the behavior of real organisms, we recorded the motion of pairs of golden shiner fish (*Notemigonus crysoleucas*), which are well established as gregarious social organisms. The previous subsection has made it clear that our experimental methods must meet three important criteria in order for a single experimental trajectory to be suitable for an information theoretic analysis. First, the fish must be geometrically confined so that the distribution of their configurational states will tend towards a stationary distribution at long times. Second, the fish must be given sufficient time to explore their environment and reach that steady state prior to the start of the experiment. Finally, the length of the experimental data collection must be long enough to ensure that there are a sufficient number of *statistically independent* datapoints to representatively sample the stationary configurational distribution of interest.

With these points in mind, we confined the fish to a roughly annular tank with an outer diameter of approximately 125.1 cm and an inner diameter of approximately 26.2 cm. In each experimental run, the two fish were given ten minutes to acclimate themselves to the tank before a high-resolution camera (mounted above) was used to record their motion for thirty minutes. A more detailed accounting of the experimental setup may be found in the Appendix A; at present we merely want to emphasize the aspects of the experimental design that are most pertinent to our statistical analysis.

To increase the likelihood of observing leader–follower behavior in the pairs of golden shiners, we attempted to create a more threatening set of external conditions both by increasing the intensity of the external lighting and by decreasing the depth of the water in the tank. Golden shiners have been shown to prefer shaded regions, presumably to avoid predators [43,44], and exposing the fish to conditions where predation seems more likely has been hypothesized to increase the attention each fish pays to its neighbors [43]. Changes in water depth also tend to invoke defensive behavior in social fish [45], causing them to remain in closer proximity to one another—an adaptive behavior that reduces individual risk within groups [46]. We found that pairs of shiners exposed to 230 lumens of external light intensity and a water depth of 4 cm were qualitatively much more active than fish tested under conditions of 200 lumens and a water depth of 8 cm, spending a much larger percentage of the recorded experiments circling the tank together. For future reference, we refer to the former set of experimental parameters as the agitated condition (due to the heightened anxiety level of the fish) and the latter set as the control condition.

The captured video data from the experiments were transcribed using the image-tracking software TRex (v1.1.3), an open-source platform that leverages computer vision and machine learning to identify and track moving entities [47]. With TRex, the posture of each individual fish was transcribed into a position time series featuring both the centroid and head locations of the fish in each frame of the video. The videos were recorded at 40 frames per second, so the time that elapsed between most consecutive datapoints in the time series was 0.025 s, although we did have to remove small numbers of points where the image-tracking software failed to clearly recognize the individual fish and consequently did not record positional data.

To take better advantage of the radial symmetry of the tank, we re-centered the positional data to shift the coordinate origin to the middle of the tank, and then we transformed the Cartesian centroid and head locations into standard polar coordinates $r_{i}$ and $\theta_{i}$, where i=0,1 indexes the two fish. The orientation (heading) of each fish, $\psi_{i}$, was computed as the angle between the positive *x*-axis and the positional vector connecting the centroid of each fish to its head. Because the tracking software sometimes had difficulty distinguishing the head of the fish from its tail, we found that the calculated heading angle would sometimes non-physically jump by nearly $180^{\circ }$ between consecutive frames. To correct for this, if $|\psi_{i}\left(t+1\right)-\psi_{i}\left(t\right)|>\pi -0.6$ radians, we set $\psi_{i}\left(t+1\right)$ equal to $\psi_{i}\left(t\right)$.

## 3. Results

### 3.1. Evidence of Follower-like Behavior

**Figure 2:**
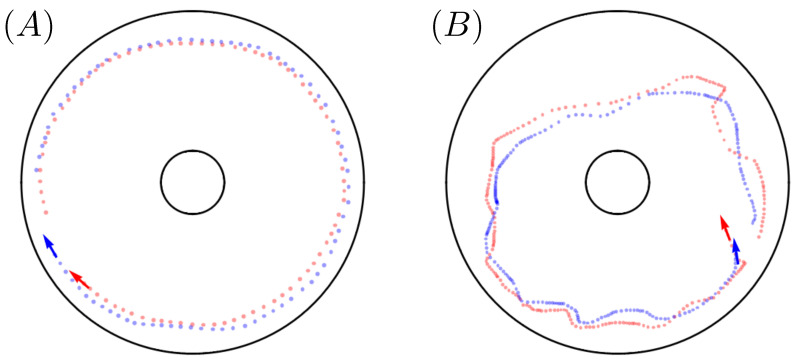
(**A**) A 10 s sample trajectory, taken from one of the agitated condition experiments, in which fish-0 (red) and fish-1 (blue) swim smooth laps about the outer wall of the annular tank. (**B**) A 25 s sample trajectory, taken from the same experimental replicate, in which the two fish swim more erratic laps about the tank.

Visualization of the trajectory data revealed that, under the agitated condition, the fish transitioned synchronously in and out of two visually distinct lap-swimming behavioral modes. In the first mode, the fish swam rapid, smooth laps around the tank, with one fish apparently following closely behind the other. In the second mode, the laps were slower and subject to more irregularity in shape due to increased radial exploration, but one fish still appeared to lead the other. Trajectory segments corresponding to these two behaviors are shown in Figure 2. Though both trajectory segments in the figure depict a single, complete lap, the smooth lap in Figure 2A is traversed much more quickly than the irregular lap in Figure 2B.

In addition to swapping between these two behavioral modes over the course of each experimental run, the fish also switched between which one of them was in the lead position, further muddying the waters as to whether a true leader–follower dynamic was at play. To more clearly delineate these intervals of apparent leader–follower behavior and distinguish them from other transient behaviors, we introduced a measure of relative fish alignment, $A_{ij}\left(t\right)$, that measured the angle between the heading vector of fish *i* and the vector connecting the centroid position of fish *i* to that of fish *j* at time *t*. Note that while, in general, hydrodynamic forces can enable a fish to drift in a direction distinct from its heading, the golden shiners in our experiments moved predominantly like self-propelled particles [48,49]. Thus the heading of each fish and its instantaneous velocity vector can be treated as roughly interchangeable for our purposes.

**Figure 3:**
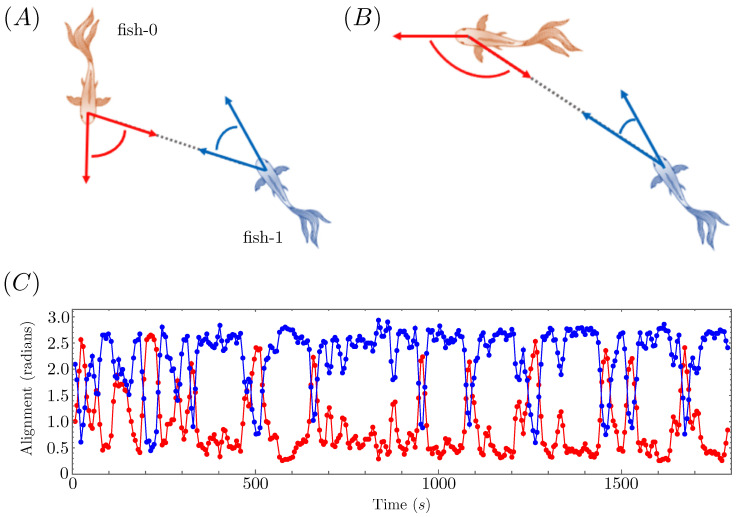
In panel (**A**), fish-0 and fish-1 are facing each other and have similar alignment angles. In panel (**B**), fish-0 leads fish-1 and fish-0 has an angle close to π, whereas fish-1 has an angle close to 0. In panel (**C**), these alignment angles are averaged over a rolling 15 s window and are plotted for both fish over the entirety of one agitated condition experiment. Note the strong polarization in the alignments over most of the experiment and that fish-1 predominantly takes the lead position ($A_{10}\gg A_{01}$ over most of the time series).

Defined in this manner, the alignment $A_{ij}$ will be small (near 0) when fish *i* is swimming directly towards fish *j* and will be large (near π) when fish *i* is swimming directly away from fish *j*. We can use this metric to identify intervals of apparent leader–follower behavior as those where $A_{ij}\approx 0$ and $A_{ji}\approx \pi$ (or vice versa). This interpretation is illustrated schematically in the top panels of Figure 3.

In the bottom panel of Figure 3, we plot the alignment angles of the two fish versus time for one of the agitated condition experiments. We used a rolling window averaging procedure to smooth out the noisiness of the raw data, so that each plotted point in the figure is actually the average alignment over the 15-s window centered at that time point. Over the course of this thirty minute experiment, the intervals of leader–follower-like behavior are those for which the two fish alignments polarize toward opposite extremes.

In this particular replicate, the alignment angles were strongly polarized one way or the other for nearly the entire experiment. Consequently, we focus on this replicate for our statistical analysis because it provides the largest quantity of useful data for interrogating whether a true leader–follower interaction can be deduced from trajectory data. Normally, cherry-picking only the best-looking data is considered intellectually dishonest; but, in our case, we are merely looking for an ideal dataset for testing our statistical methods—not to draw any generalized conclusions about golden shiner interactions. For an example of more irregular golden shiner behavior, see the alignment data plotted in Appendix A for a different experimental replicate.

### 3.2. Modeling the Data as a Simple Leader–Follower Dyad

To determine whether the observed lapping behavior of the agitated fish is driven by a genuine leader–follower dynamic, we compare the statistics of the data with the predictions of our generalized leader–follower model from Section 2.1. Since the motion of interest is approximately one-dimensional in character, we choose ${\vec{X}}_{L}\left(t\right)$ and ${\vec{X}}_{F}\left(t\right)$ as the angular coordinates $\theta_{L}\left(t\right)$ and $\theta_{F}\left(t\right)$, respectively, and assume that the two model agents are restricted to moving along a circle of fixed radius. We characterize the dynamics of the leader fish as a standard drift–diffusion process $\Delta {\vec{X}}_{L}\left(t\right)=\Omega +W_{L,\sigma }\left(t\right)$, where Ω is a fixed angular drift term and $W_{L,\sigma }\left(t\right)$ is a Gaussian white noise term with zero mean and variance $\sigma^{2}$. The error tendency of the follower fish is similarly modeled as Gaussian white noise, $\vec{\xi }\left(t\right)=W_{F,\sigma }\left(t\right)$. Thus, in parallel to Equation (3), we define our simplified fish model with the following pair of equations:

(6) $$\begin{array}{cc} \theta_{L}\left(t\right) & =\theta_{L}\left(t-1\right)+\Omega +W_{L,\sigma }\left(t\right)\\ \theta_{F}\left(t\right) & =\theta_{L}\left(t-T\right)+W_{F,\sigma }\left(t\right). \end{array}$$

In iterating the above equations, it is to be understood that the right-hand side of both equations should always be taken as modulo 2π to respect the periodicity of the circular system. We also assume the fish were both immobile prior to initialization, so that, for t<T, we can set $\theta_{L}\left(t-T\right)=\theta_{L}\left(0\right)$.

As before, *T* characterizes how long it takes the follower to observe the leader’s initial change in position and react by attempting to update its own position to that same location. The follower then continues to pursue the same trajectory as the leader (subject to its own errors in judgement), though it remains permanently lagging behind by *T* time steps due to the initial delayed reaction.

To account for the occasional changes in apparent leadership evident in the experiments, we add one final wrinkle to our toy model. At each time step, we swap the labels on the two agents with a fixed probability, α. Thus the fish in our model will switch between leader and follower roles on average every 1/α time steps. To be clear, Equation (6) describes how the leader and follower positions evolve in time, but which agent obeys which equation will change back and forth during a prolonged simulation of the model.

Our parameter choices for this model are derived from the experimental data of the agitated condition replicate in which the fish were observed to maintain the most robustly polarized alignment throughout the experiment (see Figure 3). (We include the trajectory data for this replicate in the Supplemental Information.) If we define the angular motion of fish *i* between consecutive frames of data as $\Delta \theta_{i}\left(t\right)\equiv \theta_{i}\left(t+1\right)-\theta_{i}\left(t\right)$, then we choose the angular drift of the leader, Ω, as the average of the random variable $\Delta \theta_{i}\left(t\right)$, and the noise parameter, σ, as its standard deviation. In both cases, the average is understood to be taken for both fish over the entire experimental time series.

To give our model the same dynamical resolution as the raw video data, we define the timescales 1/α and *T* in units of a simulation time step of 1/40 s. In these units, we choose α=0.00021 so that the expected number of leadership changes in thirty minutes equals the fifteen observed in the experiment, and we select T=20 (0.5 s), which is just a baseline guess derived from human visual reaction measurements.

**Figure 4:**
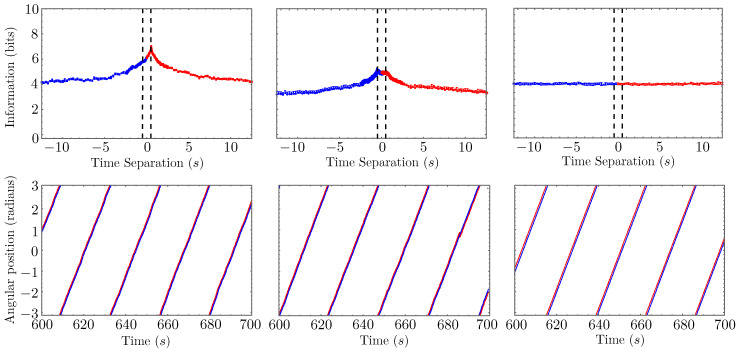
In the top three panels, we plot the time-separated mutual information $MI(\left\{\theta_{1}\left(t\right)\right\};\left\{\theta_{0}\left(t-\tau \right)\right\})$ in our one-dimensional leader–follower model for the cases (from left to right) where there is no change in leadership (α=0), occasional change in leadership (α=0.00021), and where both fish behave independently (no following behavior). The red segment of each curve emphasizes that, for positive time separations, the mutual information presumes fish-0 is the leader; the blue segment emphasizes the same for fish-1 at negative time separations. In all cases, we set Ω=0.0067 radians per time step. In the first two cases, we set σ=0.0039 radians per time step; but, in the third case, we needed to use the smaller value σ=0.00039 radians per time step to observe a trajectory that qualitatively matched those of the other two cases. The bottom three panels show segments of representative trajectories corresponding to the same three cases, with red and blue curves once again representing fish-0 and fish-1, respectively. A dashed box highlights a change in leadership in the middle panel. Note how our mutual information metric deftly distinguishes qualitatively similar path data.

Figure 4 illustrates typical behaviors for the (ensemble-sampled) mutual information $MI(\left\{\theta_{1}\left(t\right)\right\};\left\{\theta_{0}\left(t-\tau \right)\right\})$ of our model system in three distinct cases. For each calculation, we generated 300 replicate simulations, with $\theta_{0}\left(0\right)$ chosen uniformly on the interval [0,2π] and $\theta_{1}\left(0\right)$ initialized at $\theta_{0}\left(0\right)-\phi$ (modulo 2π, of course), with ϕ=0.5 for the first two cases and 0.1 for the third. Fish-0 was always initialized as the leader. We used the angular position of fish-1 at *t* = 24,000 time steps, i.e., 600 s, to ensure we were sampling from a stationary distribution in each replicate, and then, for each value of the time separation, τ, we selected the appropriate point, $\theta_{0}\left(t-\tau \right)$, from each replicate to build a dataset of 300 pairs of time points. To actually compute the mutual information, we jackknifed this set into ten randomly chosen subsets of 250 time point pairs and used the KSG algorithm on each subset. In each of the top panels of the figure, we report the mean mutual information with standard error bars.

In the top-left panel, we consider the case where α=0, and fish-0 acts as the leader for the entire time series. This results in a mutual information that monotonically decays away from a peak located at τ=0.5 s, corresponding exactly to the value of *T* chosen for the model. Note that the information does not appear to decay all the way to zero, but rather appears to asymptotically approach some finite, nonzero value. This is a consequence of the roughly periodic lapping behavior correlating the fish positions over very long timescales. A sufficiently large increase in the noise parameter, σ, washes out these long-time correlations, resulting in mutual information that fully decays towards zero.

The top-middle panel in Figure 4 plots the same mutual information for the case where α=0.00021. Now, since both fish spend time acting as leader, we see peaks emerge at both τ=0.5 and τ=−0.5 (recall that a negative time separation is equivalent to a positive time separation with the fish indices swapped). These peaks are less pronounced than in the previous case due to the stochasticity induced by the occasional leadership changes. For a sufficiently robust ensemble, the mutual information should be perfectly symmetrical about the ordinate axis since each fish has an equal opportunity to be the leader. For an ensemble with a finite number of replicates, one fish may lead a little more frequently than the other, leading to a small asymmetry. For the mutual information computed from a single trajectory time series, this asymmetry can be used to identify which fish spends more time as leader within that particular experiment. If there is also an observed asymmetry in the positions of the peaks about τ=0, then this suggests an asymmetry in the following capabilities of the agents themselves, which, while not present in our model, could certainly exist in a real animal dyad.

Finally, the top-right panel in Figure 4 considers a control case in which the fish are noninteracting (they both obey the leader dynamics of Equation (6)). In the absence of interactions, the stochastic noise causes the fish to drift apart (to an extent limited by the periodic confinement); so, to make the fairest comparison possible between this case and the previous two, we initialized the fish closer together (ϕ=0.1) and employed an order-of-magnitude smaller value of σ. The result is mutual information that is roughly constant as a function of τ; our specific choices of ϕ and σ set this constant value to something comparable to the asymptotic values observed in the other cases. An important takeaway from this comparison is that these three qualitatively distinct mutual information patterns arise from qualitatively similar model trajectories (plotted in the bottom three panels of the figure). If we had used the same values of ϕ and σ in all three cases, the rightmost MI curve would flatline at a much lower value, and the fish would be more widely separated in the corresponding trajectory.

### 3.3. Estimating the Decorrelation Timescale

For the experimental fish data, we are forced to compute the mutual information from a single time series, so we need to employ the sort of windowing procedure outlined in Section 2.2. Normally, we would choose the window size, *W*, based on the decorrelation (decay) timescale of the “true” mutual information, as computed from an ensemble of replicate trajectories, but that approach obviously does not work when only a single trajectory is available. In addition, the fact that the mutual information in our model does not appear to decay to zero with time (see Figure 4) poses another challenge to the validity of this windowing procedure. To alleviate these concerns, we propose the following procedure for computing the mutual information $MI(\theta_{1}\left(t\right);\theta_{0}\left(t-\tau \right))$ from a single time series, which we justify with our leader–follower model, where comparison with ensemble-averaged mutual information is possible.

We begin by dividing the time series for fish-1 into intervals of length *W*, for some initial choice of window size, and then we choose one time point from each interval. Rather than choose them uniformly, we treat each interval as a triangularly distributed random variable, so that we are much more likely to select time points from the middle of each interval than from the ends. To be precise, for the interval $\left[t_{i},t_{i+1}\right]$, defined so that $t_{i+1}-t_{i}=W$, we choose a single time point from the symmetric triangular distribution f(t):

(7) $$f\left(t\right)=\left\{\begin{array}{cc} 4\left(t-t_{i}\right)/W^{2}, & t\leq (t_{i}+t_{i+1})/2\\ 4\left(t_{i+1}-t\right)/W^{2}, & t>(t_{i}+t_{i+1})/2. \end{array}\right.$$

Although the resulting set of time points are only separated by *W* time steps on average when using this algorithm, we can repeat the random selection procedure to generate replicate datasets without having to reduce the size of those datasets, as we would in the jackknifing procedure discussed in the previous subsection. This is important because, in many cases, experimental trajectories are woefully short, and we want to make the most of the data available. It is also worth emphasizing that, whereas our plot of the alignment angle (see Figure 3) involves averaging over a moving temporal window, the windowing procedure we utilize for the time-separated mutual information involves no such averaging over the time points within each window.

Once we have generated our replicate datasets for $\theta_{1}\left(t\right)$, we simply take the corresponding points $\theta_{0}\left(t-\tau \right)$ from the trajectory of fish-0 to generate our sets of paired time points and use the KSG algorithm to compute the mutual information (once again reporting the average over replicate calculations with standard error bars). We then repeat the entire procedure for a larger choice of window size, and continue this search until the MI curves converge to within an acceptable tolerance.

**Figure 5:**
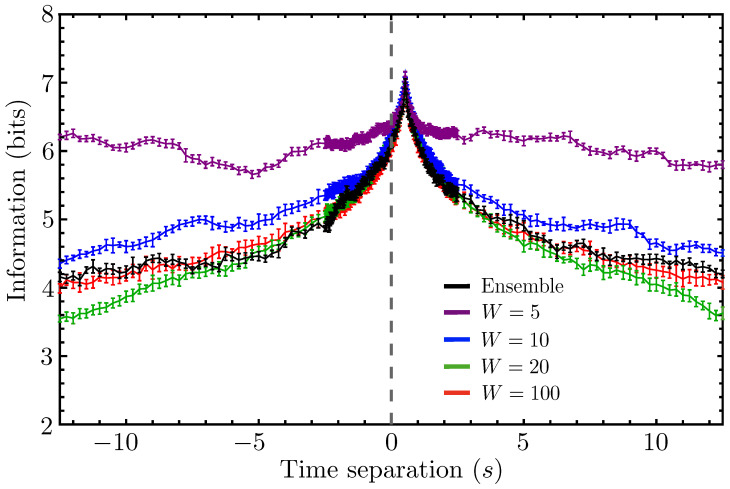
The ensemble-estimated mutual information $MI(\left\{\theta_{1}\left(t\right)\right\};\left\{\theta_{0}\left(t-\tau \right)\right\})$ is plotted for our schematic model in black and successive single-trajectory estimates, $MI(\theta_{1}\left(t\right);\theta_{0}\left(t-\tau \right))$, are plotted for different window sizes, ranging from W=5 to W=100. As expected, increasing the window between consecutively sampled pairs of time points results in convergence to the ensemble-averaged result. Even for small window sizes, however, we obtain surprisingly good convergence near the local maximum of the information, which is, in our case, the principal feature of interest.

In Figure 5, we demonstrate this method for our simplified model in the case α=0. Remarkably, even for very small window sizes of five and ten time steps (frames), we see good agreement with the ensemble-averaged result (the black curve in the figure, see also the top-left panel in Figure 4) close to where the curve peaks. These small window sizes greatly overestimate the behavior of the information at large time separations, but we often do not care about accurately reproducing this decay. In the event that we do want to accurately reproduce the entire curve, we can simply continue to increase the window size, in which case we eventually obtain convergence to the ensemble-averaged result across all timescales. Note that the red curve, which agrees quantitatively with the ensemble-averaged result, corresponds to a window size of 100 time steps, or 2.5 s, which is a reasonable estimate for the decay timescale of the black curve towards its nonzero, asymptotic limiting value.

### 3.4. Evidence of a Robust Leader–Follower Interaction

After using our phenomenological model to demonstrate how active leader–follower interactions should be expected to manifest in the mutual information shared by the time-separated angular positions of two fish (as well as how to estimate this information accurately from a single time series), we now proceed to compute the mutual information $MI(\theta_{1}\left(t\right);\theta_{0}\left(t-\tau \right))$ for the time series data collected from one of our live fish experiments. As discussed previously, we selected a replicate in which the fish synchronously swam laps around the tank for most of the experiment’s duration, with fish-1 appearing to be the dominant leader.

If the collective motion of the fish dyad in this experiment is principally driven by leader–follower-type interactions, then the mutual information computed from this dataset should exhibit a statistically significant peak at some time separation τ<0. We expect the peak to occur at a negative time separation since this corresponds, in accordance with our previously defined conventions, to the regime in which fish-1 is the leader.

**Figure 6:**
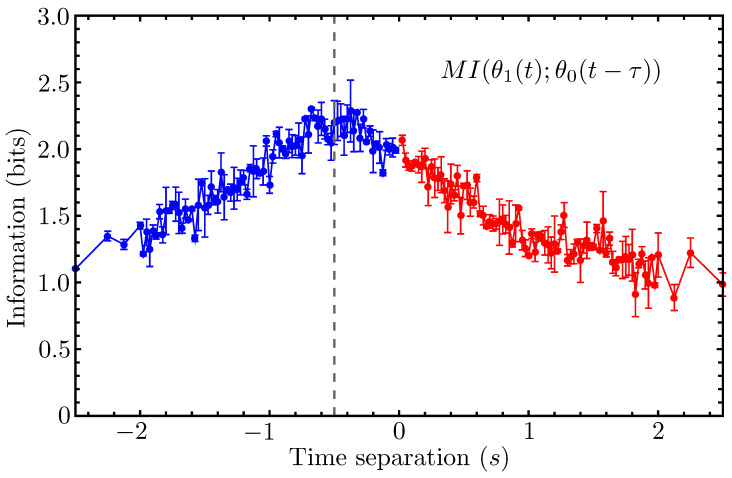
The time-separated mutual information between the angular positions of the two golden shiners, computed from a single experimental time series, is plotted for a window size W=0.5 s. A dashed vertical line marks the estimated location of the peak. This peak occurring for τ<0 implies that fish-1 is the leader during most of this time series. (The red and blue coloring has the same meaning as in the top panels of Figure 4).

In Figure 6, we plot the mutual information $MI(\theta_{1}\left(t\right);\theta_{0}\left(t-\tau \right))$ for this replicate, and the resulting curve is qualitatively similar to that plotted for our schematic model in the case of no leadership changes (see the top-left panel of Figure 4). The peak in the mutual information curve falls to the left of the ordinate axis, consistent with our prediction (based on the alignment angle time series in Figure 3) that fish-1 is the active leader for most of the experiment.

The peak in Figure 6 is much broader and noisier than that predicted by our model. This could partly be due to competing interactions that tend to obscure the leader–follower behavior; it could also be due to the follower more realistically exhibiting a stochastic reaction time with a sharply peaked average. This mean reaction time would correspond to the fixed timescale *T* in our model. To estimate the value of *T* from the experimental MI curve, it is necessary to first verify whether or not it is reasonable to treat this curve as having a single local maximum and, if it is, to then precisely estimate the location of this maximum.

**Figure 7:**
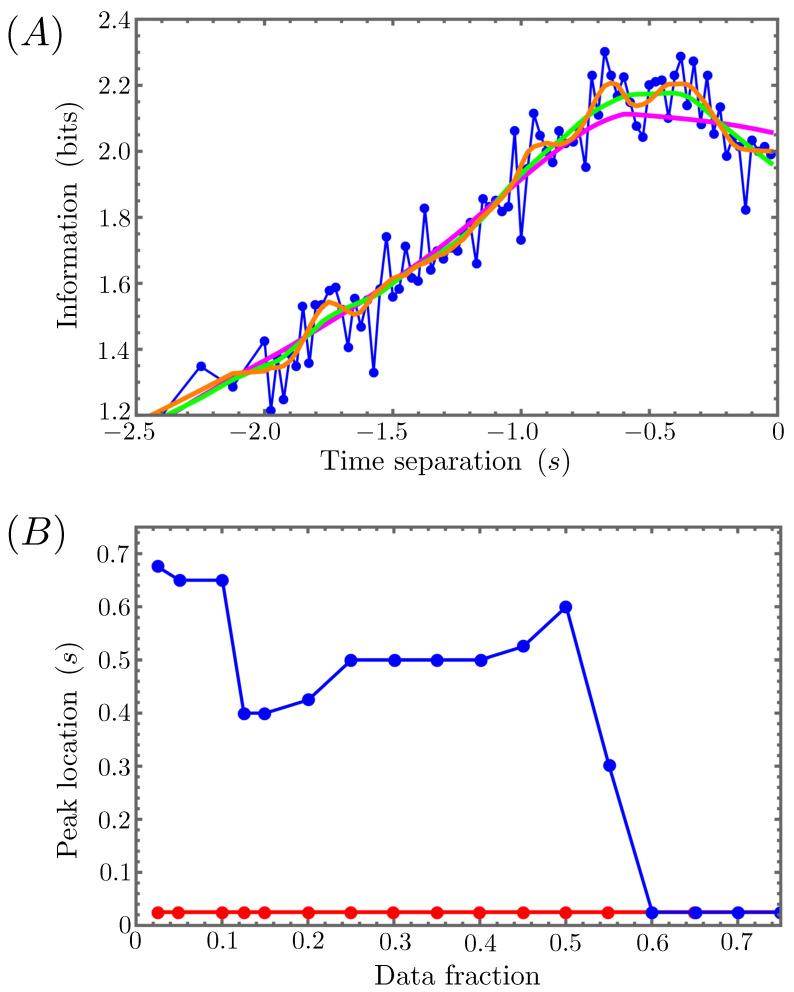
(**A**) LOESS smoothing of the τ≤0 branch of the mutual information (in blue) plotted in Figure 6 for data fractions f=0.1 (orange), f=0.2 (green), and f=0.5 (magenta). (**B**) The time separation of the peak location for this same mutual information is plotted (in blue) as a function of *f*. For a broad range of data fractions, the peak locations falls within a narrow band of time separations. The same plot in red shows that the positive time separation branch of the MI curve in Figure 6 has no peak for any data fraction.

We accomplish this by employing the standard LOESS (locally estimated scatter plot smoothing) algorithm [50]. For each point in our mutual information plot (Figure 6), LOESS takes the nearest fN datapoints, where *N* is the total number of datapoints in the plot and 0≤f≤1 is some fixed fraction of those data, and performs a locally weighted regression procedure to fit the data to a polynomial. This procedure is repeated for each datapoint in the plot, resulting in a nonparametric, systematic fitting of the overall dataset to the chosen polynomial. The important point for our purposes is that increasing the data fraction, *f*, will gradually smooth out more and more features of the raw data, and we want to ensure that the apparent peak in Figure 6 is a robust enough feature to survive smoothing over a broad range of *f* values. The top panel of Figure 7 shows the results of applying the LOESS procedure to the relevant half (τ≤0) of the mutual information curve for three different choices of *f*. The apparent peak in the curve only begins to disappear for the largest value shown (f=0.5). In the bottom panel of Figure 7, we plot the time separation corresponding to the maximal MI value for each branch of the information curve as a function of *f*, which demonstrates that, for f≤0.5, the position of the apparent peak falls consistently within a fairly narrow band of time separation values. Averaging over these values, we extract a reaction timescale of T≈0.527 s, which is not far off from our initial model choice of T=0.5 s.

## 4. Discussion

In this paper, we have demonstrated how the time-separated mutual information between the positions of two organisms can be used as a statistical hypothesis test for the existence of an asymmetric leader–follower interaction. Based on general considerations of what a leader–follower interaction should, at minimum, entail, we were able to argue that the time-separated mutual information between the relevant positional coordinates of a leader–follower dyad should exhibit a local maximum at some nonzero time separation corresponding to a signaling or communication timescale that is fundamental to the nature of the underlying interaction.

Our null hypothesis is the case of noninteracting agents who are coincidentally moving along similar trajectories; the time-separated mutual information between two such agents cannot exhibit a peak with statistical significance. Rejection of our null hypothesis does not prove the existence of a leader–follower type interaction, as there are conceivably other types of temporally retarded interactions that could produce a similar signature in the mutual information. If the time-separated mutual information curve computed for a pair of organism trajectories resembles either the upper-left or upper-center panels of Figure 4, then we can at least say that the observation is *consistent* with a dominant leader–follower interaction of the type we have modeled.

Although the time-separated mutual information enjoys a number of computational advantages over metrics, like transfer entropy, it is by no means a replacement for them in all cases. Time-separated mutual information is best employed in hypothesis testing, since it can at most say whether the time-separated correlations in a dataset match with what would be produced by a hypothesized model of directed interactions. In cases where one lacks a clear hypothesis about how information is flowing through a system, the transfer entropy can make this determination explicitly, albeit at a higher computational and more data-intensive cost.

Regardless of which information theory metric one chooses for interrogating the interactions between organisms, the experiments used to gather the requisite trajectory data must be carefully designed. To be optimal for an information theory analysis, the organisms of interest should be confined to a finite volume, and they must be permitted to explore that volume long enough to achieve a stationary distribution of organism positions. Data collection must be performed for as long a time as possible as well, since a potentially large proportion of the data will have to be discarded to ensure that each pair of time points considered can be treated as independent, uncorrelated samples.

Generally speaking, these constraints may be a tall order to implement in many systems of interest, but we have succeeded in conducting a set of experiments on pairs of golden shiners that fulfill all the desired criteria. Analyzing the trajectory data extracted from one of these experiments, we found a mutual information curve that qualitatively matched the predictions of our simple model, and from this we were able to estimate the signaling timescale of the apparent leader–follower interaction driving the synchronous lap-swimming behavior of the fish. Given the similarity of this timescale to human visual reaction times (and given the observed similarities between human and piscine vision), we can even speculate that a golden shiner tracks the motion of its confederates principally through visual cues (as opposed, for example, to sensing through the hydrodynamic fluctuations propagated by confederate motion).

## Figures and Tables

**Figure A1:**
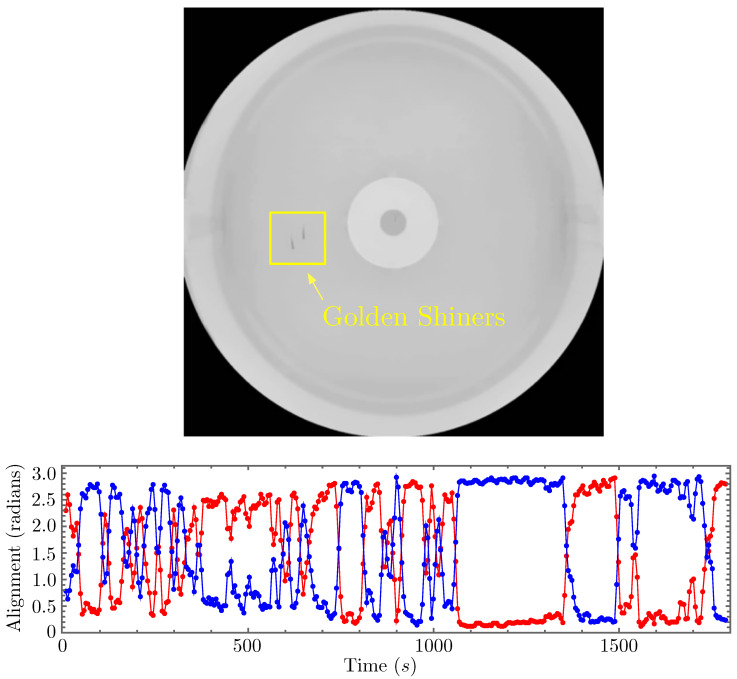
Top panel: raw video frame from one of the golden shiner experiments showing the annular tank and the fish dyad. Bottom panel: a 15 s rolling window average of the alignment angles of fish-0 (in red) and fish-1 (in blue) computed over the course of an agitated condition replicate experiment different than that used throughout the rest of the paper. While the lapping behavior in this replicate is not quite as smooth and consistent as that shown in Figure 3, long intervals of apparent leader–follower behavior can still be observed.

## Data Availability

All the codes and algorithms used for this work may be found in the appendices of author K. Daftari’s thesis, available here (accessed on 4 September 2024): https://cdr.lib.unc.edu/concern/dissertations/8p58pq754. The experimental dataset used in our information theory analysis will be made available by the authors upon request.

## Supplementary Materials

The following supporting information can be downloaded at: https://www.mdpi.com/article/10.3390/e26090775/s1.

## Appendix A. Experimental Details

Our collaborators at the Cognitive Ecology and Behavioral Engineering Laboratory (BEL) of the U.S. Army Corp of Engineer’s Engineering Research and Development Center (ERDC) in Vicksburg, Mississippi performed the data collection and experimental management. Outside of experimentation, four hundred juvenile golden shiners were housed in an indoor holding tank with fluorescent lights on a 12h light/dark cycle. Fish were fed once daily ad libitum, after experimental individuals had been selected. To ensure a sterile environment, water quality measurements were taken daily, including temperature, dissolved oxygen, pH, conductivity, and oxidation-reduction potential (ability of water to break down contaminants). Additional weekly tests were performed to measure ammonia and nitrate content as well as carbonate hardness.

Experimental pairs were selected randomly and taken to the experimental tank in a separate temperature-controlled room. An aerial view of the annular tank setup is shown in the top panel of Figure A1. The inner diameter of the tank measured 26.2 cm and the outer diameter measured 125.1 cm. Once (unfed) experimental pairs were transferred to the tank, the fish were left undisturbed for 10 min to acclimate to the new environment. After the acclimation period, video data were recorded using a Basler Boost Monochrome high resolution camera for a period of 30 min at a frame rate of 40 frames per second and a resolution of 2912 × 2750 pixels. The still images (frames) of the video data were converted into a continuous video format using the ffmpeg library. Four replicate experiments were performed in the control setting, and five experiments were performed in the agitated setting. At the conclusion of each 30-min experiment, fish weights and lengths were measured before euthanization.

While we only applied our statistical analysis to the agitated replicate featuring the most consistent lapping behavior, the trajectories observed in that replicate were not behavioral outliers, as one can see from the bottom panel of Figure A1, in which the alignment angles of the two fish are plotted for a different agitated replicate than that used for our analysis (compare this plot with that of Figure 3).

## References

[1] Biro D.D., Sumpter J., Meade J., Guilford T. (2006). From Compromise to Leadership in Pigeon Homing. Curr. Biol..

[2] Collignon B., Detrain C. (2010). Distributed leadership and adaptive decision-making in the ant *Tetramorium caespitum*. Proc. R. Soc. B.

[3] Bousquet C.A.H., Manser M.B. (2011). Resolution of experimentally induced symmetrical conflicts of interest in meerkats. Anim. Behav..

[4] Ginelli F., Peruani F., Pillot M.H., Chaté H., Theraulaz G., Bon R. (2015). Intermittent Collective Dynamics Emerge from Conflicting Imperatives in Sheep Herds. Proc. Natl. Acad. Sci. USA.

[5] Hodgkin L.K., Symonds M.R.E., Elgar M.A. (2017). Leadership through knowledge and experience in a social sawfly. Anim. Behav..

[6] Sasaki T., Mann R.P., Warren K.N., Herbert T., Wilson T., Biro D. (2017). Personality and the collective: Boldhoming pigeons occupy higher leadershipranks in flocks. Philos. Trans. R. Soc. B.

[7] Webster M.M. (2017). Experience and motivation shape leader–follower interactions in fish shoals. Behav. Ecol..

[8] Bevan P.A., Gosetto I., Jenkins E.R., Barnes I., Ioannou C.C. (2018). Regulation between personality traits: Individual social tendencies modulate whether boldness and leadership are correlated. Proc. R. Soc. B.

[9] King A.J. (2010). Follow me! I’m a leader if you do; I’m a failed initiator if you don’t?. Behav. Process..

[10] Strandburg-Peshkin A., Papageorgiou D., Crofoot M.C., Farine D.R. (2017). Inferring influence and leadership in moving animal groups. Philos. Trans. R. Soc. B.

[11] Orange N., Abaid N. (2015). A transfer entropy analysis of leader-follower interactions in flying bats. Eur. Phys. J. Spec. Top..

[12] Porfiri M. (2018). Inferring Causal Relationships in Zebrafish-Robot Interactions through Transfer Entropy: A Small Lure to Catch a Big Fish. Anim. Behav. Cogn..

[13] Valentini G., Mizumoto N., Pratt S.C., Pavlic T.P., Walker S.I. (2020). Revealing the structure of information flows discriminates similar animal social behaviors. eLife.

[14] Xie W., Gao D., Lee E.W. (2022). Detecting Undeclared-Leader-Follower Structure in Pedestrian Evacuation Using Transfer Entropy. IEEE Trans. Intell. Transp. Syst..

[15] Schreiber T. (2000). Measuring Information Transfer. Phys. Rev. Lett..

[16] Crosato E., Jiang L., Lecheval V., Lizier J.T., Wang X.R., Tichit P., Theraulaz G., Prokopenko M. (2018). Informative and misinformative interactions in a school of fish. Swarm Intell..

[17] Kaiser A., Schreiber T. (2002). Information transfer and continuous processes. Phys. D.

[18] Spinney R.E., Prokopenko M., Lizier J.T. (2017). Transfer entropy in continuous time, with applications to jump and neural spiking processes. Phys. Rev. E.

[19] Papana A., Papana-Dagiasis A., Siggiridou E. (2020). Shortcomings of Transfer Entropy and Partial Transfer Entropy: Extending Them to Escape the Curse of Dimensionality. Int. J. Bifurc. Chaos.

[20] Kraskov A., Stögbauer H., Grassberger P. (2004). Estimating Mutual Information. Phys. Rev. E.

[21] Lord W.M., Sun J., Ouellette N.T., Bollt E.M. (2016). Inference of Causal Information Flow in Collective Animal Behavior. IEEE Trans. Mol. Biol. Multi-Scale Commun..

[22] Lahiri S., Nghe P., Tans S.J., Rosinberg M.L., Lacoste D. (2017). Information-theoretic analysis of the directional influence between cellular processes. PLoS ONE.

[23] Shaffer I., Abaid N. (2020). Transfer Entropy Analysis of Interactions between Bats Using Position and Echolocation Data. Entropy.

[24] Zhu J., Bellanger J.J., Shu H., Jeannès R.L.B. (2015). Contribution to Transfer Entropy Estimation via the k-Nearest-Neighbors Approach. Entropy.

[25] Darmon D., Rapp P.A. (2017). Specific transfer entropy and other state-dependent transfer entropies for continuous-state input-output systems. Phys. Rev. E.

[26] Rozo A., Morales J., Moeyersons J., Joshi R., Caiani E.G., Borzée P., Buyse B., Testelmans D., Huffel S.V., Varon C. (2021). Benchmarking Transfer Entropy Methods for the Study of Linear and Nonlinear Cardio-Respiratory Interactions. Entropy.

[27] Sun J., Bollt E.M. (2014). Causation entropy identifies indirect influences, dominance of neighbors and anticipatory couplings. Phys. D Nonlinear Phenom..

[28] Weilenmann M., Colbeck R. (2017). Analysing causal structures with entropy. Proc. R. Soc. A Math. Phys. Eng. Sci..

[29] De Lellis P., Marín M.R., Porfiri M. (2022). Inferring directional interactions in collective dynamics: A critique to intrinsic mutual information. J. Phys. Complex..

[30] Endo W., Santos F.P., Simpson D., Maciel C.D., Newland P.L. (2015). Delayed mutual information infers patterns of synaptic connectivity in a proprioceptive neural network. J. Comput. Neurosci..

[31] Li S., Xiao Y., Zhou D., Cai D. (2018). Causal inference in nonlinear systems: Granger causality versus time-delayed mutual information. Phys. Rev. E.

[32] Yang B., Zhang W., Wang H., Song C., Chen Y. (2016). TDSDMI: Inference of time-delayed gene regulatory network using S-system model with delayed mutual information. Comput. Biol. Med..

[33] Zeng Y., He Y., Zheng R., Li M. (2023). Inferring single-cell gene regulatory network by non-redundant mutual information. Brief. Bioinform..

[34] Ji C., Ma F., Wang J., Wang J., Sun W. (2021). Real-time industrial process fault diagnosis based on time delayed mutual information analysis. Processes.

[35] Albers D.J., Hripcsak G. (2012). Estimation of time-delayed mutual information and bias for irregularly and sparsely sampled time-series. Chaos Solitons Fractals.

[36] Bellomo N., Egidi M. (2024). From Herbert A. Simon’s legacy to the evolutionary artificial world with heterogeneous collective behaviors. Math. Model. Methods Appl. Sci..

[37] Nguyen G.P., Wittmann R., Löwen H. (2021). Active Ornstein–Uhlenbeck model for self-propelled particles with inertia. J. Phys. Condens. Matter.

[38] Qi T., Lin J., Ouyang Z. (2022). Hydrodynamic behavior of self-propelled particles in a simple shear flow. Entropy.

[39] Borzi A., Wongkaew S. (2015). Modeling and control through leadership of a refined flocking system. Math. Model. Methods Appl. Sci..

[40] Wilmer A., de Lussanet M., Lappe M. (2012). Time-delayed mutual information of the phase as a measure of functional connectivity. PLoS ONE.

[41] Albers D.J., Hripcsak G. (2012). Using time-delayed mutual information to discover and interpret temporal correlation structure in complex populations. Chaos Interdiscip. J. Nonlinear Sci..

[42] McMillen P., Walker S.I., Levin M. (2022). Information Theory as an experimental tool for integrating disparate biophysical signaling modules. Int. J. Mol. Sci..

[43] Ward A.J.W., Sumpter D.J.T., Couzin I.D., Hart P.J.B., Krause J. (2008). Quorum decision-making facilitates information transfer in fish shoals. Proc. Natl. Acad. Sci. USA.

[44] Berdahl A., Torney C.J., Ioannou C.C., Faria J.J., Couzin I.D. (2013). Emergent Sensing of Complex Environments by Mobile Animal Groups. Science.

[45] Bode N.W.F., Faria J.J., Franks D.W., Krause J., Wood A.J. (2010). How perceived threat increases synchronization in collectively moving animal groups. Proc. R. Soc. B Biol. Sci..

[46] Hamilton W. (1971). Geometry for the selfish herd. J. Theor. Biol..

[47] Walter T., Couzin I.D. (2021). TRex, a fast multi-animal tracking system with markerless identification, and 2D estimation of posture and visual fields. eLife.

[48] ten Hagen B., van Teeffelen S., Löwen H. (2011). Brownian motion of a self-propelled particle. J. Phys. Condens. Matter.

[49] Caprini L., Marini Bettolo Marconi U. (2021). Inertial self-propelled particles. J. Chem. Phys..

[50] Cleveland W.S. (1979). Robust locally weighted regression and smoothing scatterplots. J. Am. Stat. Assoc..

